# Consumer Perceptions of Wearable Technology Devices: Retrospective Review and Analysis

**DOI:** 10.2196/17544

**Published:** 2020-04-20

**Authors:** Kimberly P L Chong, Julia Z Guo, Xiaomeng Deng, Benjamin K P Woo

**Affiliations:** 1 University of California, Los Angeles Sylmar, CA United States

**Keywords:** wearable technology devices, Fitbit, Amazon, sleep

## Abstract

**Background:**

Individuals of all ages are becoming more health conscious, and wearable technology devices (eg, Fitbit and Apple Watch) are becoming increasingly popular in encouraging healthy lifestyles.

**Objective:**

The aim of this paper was to explore how consumers use wearable devices.

**Methods:**

A retrospective review was done on the top-rated verified purchase reviews of the Fitbit One posted on Amazon.com between January 2014 and August 2018. Relevant themes were identified by qualitatively analyzing open-ended reviews.

**Results:**

On retrieval, there were 9369 reviews with 7706 positive reviews and 1663 critical reviews. The top 100 positive and top 100 critical comments were subsequently analyzed. Four major themes were identified: sleep hygiene (“charts when you actually fall asleep, when you wake up during the night, when you're restless--and gives you a cumulative time of “actual sleep” as well as weekly averages.”), motivation (“25 lbs lost after 8 months – best motivator ever!”), accountability (“platform to connect with people you know and set little competitions or group…fun accountability if you set a goal with a friend/family.”), and discretion (“able to be clipped to my bra without being seen.”). Alternatively, negative reviewers felt that the wearable device’s various tracking functions, specifically steps and sleep, were inaccurate.

**Conclusions:**

Wearable technology devices are an affordable, user-friendly application that can support all individuals throughout their everyday lives and potentially be implemented into medical surveillance, noninvasive medical care, and mobile health and wellness monitoring. This study is the first to explore wearable technology device use among consumers, and further studies are needed to examine the limitless possibilities of wearable devices in health care.

## Introduction

Wearable technology devices are applications for monitoring and tracking fitness-related metrics such as steps taken, distance walked or ran, and calories consumed. The World Health Organization recommends that adults aged 18 to 64 years should do at least 150 minutes of moderate-intensity aerobic physical activity throughout the week, at least 75 minutes of vigorous-intensity aerobic physical activity throughout the week, or an equivalent combination of both to improve cardiorespiratory and muscular fitness and bone health, and reduce the risk of chronic diseases and depression [1]. Wearable devices provide an application for individuals to meet those guidelines. These devices are becoming increasingly popular due to advancements in technology and the public’s increased health consciousness. Wearable technology was determined to be the top fitness trend worldwide in 2016 and 2017, and continues to be number 1 in the top 20 worldwide fitness trends for 2019 [2-4]. Because of this growing trend, it becomes important to determine wearable devices’ potential to be used, not only in fitness, but in various aspects of health care. With continual improvements, wearable technology devices offer a wide array of functions, including the ability to track steps, calories consumed and burned, floors climbed, sleep, and heart rate, as well as providing silent alarms. By using wearable devices, individuals are becoming more health conscious, thus, enabling them to take control of their own health. Therefore, we conducted an initial review of wearable technology devices, based on reviews posted on Amazon.com, to obtain an initial understanding of how this type of technology might be further implemented in health care.

## Methods

A retrospective review of the top-rated verified purchase reviews of the Fitbit One posted on Amazon.com between March 2013 and August 2018 was conducted. The text of the top 100 most helpful positive and the top 100 most critical comments or reviews as rated by consumers were analyzed. To analyze the responses gathered on Amazon.com, the author used qualitative analysis to identify relevant themes. This research was deemed exempt from the University of California, Los Angeles Institutional Review Board.

## Results

### Findings

On initial retrieval, 9369 reviews of the Fitbit One on Amazon.com were submitted over the 5-year period previously mentioned. A total of 200 reviews were subsequently analyzed, 100 positive and 100 critical comments. Four major themes were identified: sleep hygiene, motivation, accountability, and discretion. Textbox 1 provides themes along with representative quotes. Demographic data of those who posted reviews were not available, unless the reviewer provided this information within their review. For example, one reviewer wrote “at the age of 75+…”.

**Themes of wearable technology device uses and representative quotes.**

**Sleep hygiene**

- “I wondered why I was so tired when I got up in the morning. The fitbit really does track my sleep patterns. I found that I was awake numerous times (it even tell you exactly what times you are awake) and it shows when you are restless (it also shows those exact times). I found that about 1/2 of the time I am asleep I am not getting actual sleep time.”
- “Another feature is the sleep tracking; having sleep apnea, I can check my sleep quality from both my CPAP machine and the One. Some nights I wake up quite a bit, and the graphs reflect this.”

**Motivation**

- “…this works! It's easy to check during the day to keep you on target. In fact I actually WANT to check it, to see my progress. My goal, of course is 10,000 steps a day. Thanks to my Fitbit One, I know I'm going to get there on a regular basis. It gets me out and walking and keeps me moving. I am constantly challenging myself. I finally found something that motivates me to exercise.”
- “If you’re highly competitive getting one of these then competing with your friends on it might work…help keep you motivated.”

**Accountability**

- “…held me accountable and reminded me to get moving. After sitting at a desk the majority of the day, I would notice my steps were low and I would go for a walk/run or take an exercise class to reach my goal of 10,000 steps.”
- “there's a platform to connect with people you know and set little competitions or group goals…fun accountability if you set a goal with a friend/family.”

**Discretion**

- “…because I didn't want to wear a wristband all day since I am constantly typing I knew it would drive me nuts… I really like this little guy I clip it to my bra… don't even notice it is there throughout the day.”
- “The wristbands are all the rage, I know, but this little guy has a key advantage for me -- it clips onto my bra. Honestly, that is my #1 favorite feature. I don't have to worry about what it looks like because no one can see it. Ta-da! Out of sight, out of mind; no need to worry about whether a silicone bracelet goes with your outfit.”

### Theme 1: Sleep Hygiene

Sleep was one of the more commonly cited uses. Fitbit One users reported using the sleep tracking function, which records the duration of sleep and when users are “asleep”, “restless”, and “awake”. One positive review stated, “I wondered why I was so tired when I got up in the morning…found that about ½ of the time I am asleep I am not getting actual sleep time.” Another positive review stated, “The sleep graph…encourages…to improve their sleep habits.” Some parents even found the Fitbit One useful in alerting them on when to take their child to see a physician due to the recordings of the child’s sleep patterns.

When my son tracked his sleep patterns, he found that he was averaging only about 2 hours of sleep a night. He has been diagnosed with sleep apnea and is receiving treatment.

Users also enjoyed the silent alarm function, which allows the tracker to wake users up with a quiet vibration. One positive review stated, “ love the silent alarms – I was skeptical that a little thing buzzing on my wrist would wake me up – but it works great and my husband appreciates how quiet it is.”

### Theme 2: Motivation

Several users discussed how they had been able to use the Fitbit One to continue to motivate themselves to stay active and continue to set goals as they strive to live healthier lifestyles. They have been able to successfully integrate the tracking functions into their daily routines, including steps and activities, calories burned, floors climbed, weight, water consumption, food intake, and exercise. One person wrote, “Since you become aware of what you are doing, it is self-motivating to adjust and add more activity.” Another person stated:

We all know we have to be more active, but this little tracker reminds you, with facts and real data, how much real activity we are doing every day, and that is an eye-opener and a great motivator. I want to beat the tracker so I walk to get to the 10,000 steps.

Not only is it a good tool for self-motivation, but the ability to form a personal network on the app with friends and family allows users to help motivate each other. One user wrote:

…the community is fun. I have a number of friends, family, and co-workers who have devices and we issue challenges a lot…definitely a nice way to get support.

By using the tracking functions, the Fitbit One also helps users along their weight loss journey. Many reviews boasted about how using a wearable device helped them lose weight.

I’ve integrated my One into my everyday life, and I’m the happiest because of it. It’s been just 2 ½ months since my first weight at the doctor’s office. As of yesterday, I’ve dropped 24 pounds. And I did so naturally.

### Theme 3: Accountability

Several reviewers reported that they had been using the Fitbit One to keep them accountable. One user stated, “I do see myself improving my activity level from this as well as my eating habits, it makes one accountable for one’s actions.” Another wrote:

All I want right now is a reminder-helper-assistant- cool gadget, that helps me realize how much activity I'm doing or not doing, pats me in the back when I do something good and holds me accountable when I get lazy, all in a nice, cool, fun and easy way… I'm feeling accomplished, energetic and in control, and that to me is absolutely worth it.

### Theme 4: Discretion

Interestingly, discretion was a feature of the Fitbit One that many reviewers commented about. One positive review stated, “…didn’t want everyone I came across to see that I was tracking my steps.” Also, because this specific device allows the options of wearing it on one’s wrist, clipped to clothing, or even tucked into a pocket, many users wrote positive reviews about this flexibility. One user wrote:

this little guy has a key advantage for me – it clips onto my bra. Honestly, that is my #1 favorite feature. I don’t have to worry about what it looks like because no one can see it…no need to worry about whether a silicone bracelet goes with your outfit.

Others did not want to be constantly preoccupied with checking their trackers: “I chose the clip-on style vs. the wristband because I didn’t want to be hyper-aware of my steps and then start obsessing over them.”

As expected, not all customer reviews were positive. Although the step tracker “seems pretty accurate” according to multiple users, some do complain of inaccuracies especially with the floors climbed tracker. One critical review stated:

The reliability of the count is not great but is acceptable when it comes to counting steps. When it comes to counting the floors climbed…found it ridiculously inaccurate. For example, a few times it indicated that I had climbed 10 or 15 floors when I had not left (my single floored) apartment.

Some users also found the sleep tracker to be inaccurate. One user commented, “When it came to tracking my sleep, it suggested that I had minutes of sleep after I slept throughout the night.” Other users also critiqued the possible inaccuracies of the sleep tracker due to the wrist Velcro band falling off while they were sleeping. Some critical reviews also pointed out that the Fitbit One did not contain a heart rate monitor and was not waterproof, but newer models have added these features and more.

## Discussion

### Principal Results

Our study suggests that wearable technology devices could be helpful tools to provide functional and social stimulation to individuals of all ages and support healthy lifestyles. Four major themes were discovered, including sleep hygiene, motivation, accountability, and discretion. This research looked at current real-world use of wearable technology devices as it examined “top rated, verified purchase” reviews of the Fitbit One. Compared with research tools like Actigraph, these devices are considered less accurate for some measurements [5,6]. However, they are generally less invasive, cheaper, more user-friendly, and more fashionable, as well as offering more functionality. The relative accessibility and affordability of wearable technology devices provides an opportunity to expand nationally and even worldwide. Multiple users commented on how Fitbit was a “lifesaver”, from alerting a mother to bring her son to a physician to individuals who are obese who finally found a way to persevere with their weight loss journey. Currently, studies are being done with wearable devices and different disease states like sleep and arrhythmia. Although these studies show that the devices still have insufficient accuracy for clinical settings, solving technical issues and continuing to optimize clinically oriented features could make them available for use in clinical practice in a nondistant future [7-10]. Although Fitbit may not currently replace diagnostic tools like polysomnography for obstructive sleep apnea or Holter monitoring for atrial fibrillation, wearable devices may provide a cost-effective opportunity to alert individuals to see a physician earlier and prevent life-threatening complications.

### Limitations

There were some limitations to this study. First, this was a retrospective review of comments that were only posted on Amazon.com. The purpose of these reviews was to provide opinions to other shoppers interested in purchasing the Fitbit One. There is a potential bias when only using posted reviews from selected customers since many more devices were sold than there were reviews. The strength of the study was that the comments were obtained from real-world users, but further research is needed on the actual use of wearable devices and potential applications in medical surveillance, noninvasive medical care, and mobile health and wellness monitoring [11-14]. Second, these reviews were limited to one specific company and technology, but studies have shown no significant difference in tracking when compared to other wearable devices like the Apple Watch, Jawbone, and Mi Band [15]. Third, we were unable to obtain specific demographic information on the users, so further research would be helpful in determining the different benefits within various age groups with the use of wearable technology devices. Fourth, there are benefits and risks to using wearable devices and further research should be done to identify these risks [16]. Nonetheless, valuable information can be obtained from this exploratory analysis to help guide in future research.

### Future Research

Future research could investigate the barriers to the use of wearable technology devices in individuals of all ages and additional features that would encourage more use of these products. Possible improvements include making modifications for more precise tracking of steps taken and floors climbed, and sleep, as well as the use of a heart rate monitor and a longer lasting and stable material that is water resistant. This information could be valuable to the companies that develop the wearable devices for future upgrades to better serve customers. Thus, they could provide the mutually beneficial opportunity for increased sales and further enhancement of healthy living worldwide. In addition, further research is needed to determine how wearable technology devices can be implemented in health care. Research that is disease specific (eg, obstructive sleep apnea, atrial fibrillation, depression, obesity) would also be helpful in examining how best to expand the use of wearable devices. For example, sleep tracking can be used to diagnose obstructive sleep apnea; the heart rate monitor and possible advancing of monitoring heart activity can be used for arrhythmias; step and activity tracking can help physicians track individuals at risk for depression; food log and calorie trackers can track individuals at risk for conditions such as obesity, diabetes, hypercholesterolemia, or anorexia nervosa; stress level monitoring can track anxiety and panic attacks; and blood pressure monitoring can help those at risk for hypertension.

### Conclusions

In conclusion, wearable technology devices show great promise in many aspects of health care, from fitness to health and wellness monitoring to possible future diagnostic tools. The wide range of reported quality of life improvement that the wearable technology device already provides (sleep hygiene, motivation, accountability, and discretion) are just a few of the various possibilities for companies to further develop this technology to impact lives worldwide.
